# Unexplained hypercapnia with normal pulmonary evaluation in a patient receiving semaglutide: a diagnostic challenge

**DOI:** 10.1210/jcemcr/luag121

**Published:** 2026-04-22

**Authors:** Sai Prasad, Aarushi Ahuja, Laxmansa C Katwa, Shreya Sharma, Vidhi Parmar, Shaily Agrawal

**Affiliations:** Department of Internal Medicine, S. Nijalingappa Medical College, Bagalkote, Karnataka 587102, India; Department of Internal Medicine, Maulana Azad Medical College, New Delhi, Delhi 110002, India; Department of Physiology, East Carolina University Brody School of Medicine, Greenville, NC 27834, USA; Department of Internal Medicine, Hamdard Institute of Medical Sciences and Research, New Delhi, Delhi 110062, India; Department of Internal Medicine, D. Y. Patil Medical College, Navi Mumbai, Maharashtra 400706, India; Department of Internal Medicine, Shri M. P. Shah Government Medical College, Jamnagar, Gujarat 361006, India

**Keywords:** semaglutide, glucagon-like peptide-1 receptor agonists, hypercapnia, obesity, respiratory depression

## Abstract

Glucagon-like peptide-1 (GLP-1) receptor agonists are widely used for the management of obesity; however, respiratory complications are rarely documented. A 40-year-old person without diabetes with obesity (body mass index [BMI] 36 kg/m^2^) developed excessive daytime sleepiness and morning headaches four weeks after semaglutide dose escalation to 1 mg per week. Arterial blood gas analysis revealed respiratory acidosis (pH 7.33 [reference range (RR): 7.35-7.45], partial pressure of arterial carbon dioxide [PaCO_2_] 56 mmHg [RR: 35-45 mmHg]) with a normal alveolar-arterial (A–a) gradient. A comprehensive evaluation excluded conventional causes of hypercapnia. Overnight capnographic monitoring using transcutaneous carbon dioxide (CO_2_) demonstrated sustained nocturnal hypercapnia without apneic events (Apnea-Hypopnea Index [AHI] < 5 events/hour), suggesting impaired central ventilatory drive rather than sleep-disordered breathing. Following semaglutide discontinuation and temporary noninvasive ventilation (NIV), symptoms resolved within 10 days. Arterial blood gas levels normalized at 3 weeks (pH 7.38, PaCO_2_ 44 mmHg). The patient remained asymptomatic at the 3-month follow-up. This case demonstrates a previously uncharacterized association between semaglutide and reversible central respiratory depression. Clinicians should remain vigilant for unexplained hypercapnia in individuals receiving GLP-1 receptor agonists, particularly following dose escalation.

## Introduction

Glucagon-like peptide-1 (GLP-1) receptor agonists have emerged as cornerstones in managing type 2 diabetes and obesity, with semaglutide demonstrating substantial weight loss and cardiovascular benefits [1-4]. The medication enhances glucose-dependent insulin secretion, suppresses glucagon release, delays gastric emptying, and modulates appetite through central nervous system pathways [5]. The safety profile has been extensively characterized, with gastrointestinal adverse effects (nausea, vomiting, diarrhea) being most common [6]. Rare associations, including pancreatitis, cholelithiasis (likely related to rapid weight loss rather than the drug itself), and temporary worsening of diabetic retinopathy (associated with rapid glycemic improvement rather than a direct drug effect), have been reported [7, 8]. However, the potential respiratory effects remain largely unexplored. GLP-1 receptors are widely distributed in the central nervous system, particularly in the nucleus tractus solitarius, area postrema, and ventrolateral medulla—regions involved in autonomic and respiratory regulation—as well as in lung tissue and glandular epithelium [9-11]. Although the brainstem contains respiratory control centers, the interaction between GLP-1 receptor activation and ventilatory drive has not been systematically investigated clinically.

We present a case of unexplained hypercapnia with respiratory acidosis in a person receiving semaglutide therapy, in whom comprehensive evaluation excluded all traditional causes of hypoventilation.

## Case presentation

A 40-year-old man without diabetes presented with excessive daytime somnolence of 10 days' duration, associated with morning headaches for one week and intermittent hunger sensations at rest for five days. He denied any history of dyspnea, cough, wheezing, fever, chest pain, orthopnea, or paroxysmal nocturnal dyspnea.

The patient had class II obesity with a body mass index (BMI) of 36 kg/m^2^ (height, 170 cm; weight, 104 kg). He had been receiving semaglutide for obesity management, initiated at 0.25 mg/week for the first four weeks per standard escalation protocol. The dose was then escalated to 0.5 mg/week for an additional four weeks. At that point, based on the patient's response and clinical assessment, the treating physician escalated the dose to 1 mg/week. This escalation occurred after only four weeks at the 0.5 mg dose, which is consistent with the minimum interval recommended in standard dosing guidelines (escalation no sooner than every four weeks); however, the standard protocol permits remaining at 0.5 mg for longer if tolerability is a concern. Semaglutide had therefore been ongoing for twelve weeks total at the time of presentation, with the 1 mg/week dose initiated four weeks before symptom onset.

The patient had no prior exposure to other incretin-based therapies, including dipeptidyl peptidase-4 (DPP-4) inhibitors, tirzepatide, or other GLP-1 receptor agonists. There was no history of chronic lung disease, obstructive sleep apnea (OSA), neuromuscular disorders, cardiac disease, or prior cerebrovascular events. The patient denied alcohol consumption, smoking, and the use of opioids, sedatives, or other respiratory depressants. His current medications included only semaglutide; no other medications, supplements, or over-the-counter preparations were used. Glycated hemoglobin (HbA1c) level was 5.4% (reference range [RR]: <5.7%), consistent with a status without diabetes.

## Diagnostic assessment

On presentation, the patient was conscious and well-oriented to time, place, and person. Vital signs included a respiratory rate of 14 breaths per minute, oxygen saturation of 96% on room air, heart rate of 82 beats per minute, and blood pressure of 128/80 mmHg. Chest examination revealed clear lung fields bilaterally. Cardiovascular and neurological examinations were unremarkable. There was no peripheral edema or clinical evidence of neuromuscular weakness.

Arterial blood gas analysis on room air demonstrated a pH of 7.33 (RR: 7.35-7.45), PaCO_2_ of 56 mmHg (RR: 35-45 mmHg), partial pressure of arterial oxygen (PaO_2_) of 78 mmHg (RR: 80-100 mmHg), bicarbonate (HCO_3_^−^) of 29 mmol/L (RR: 22-26 mmol/L), and an A–a gradient within age-adjusted normal limits, suggesting preserved gas exchange without ventilation-perfusion mismatch.

Routine hematological investigations revealed hemoglobin 14.6 g/dL (SI: 146 g/L) (RR: 13.5-17.5 g/dL [SI: 135-175 g/L]), total leukocyte count 7400/μL (SI: 7.4 × 10^9^/L) (RR: 4000–11 000/μL [SI: 4.0-11.0 × 10^9^/L]), and platelet count 260 000/μL (SI: 260 × 10^9^/L) (RR: 150 000–450 000/μL [SI: 150-450 × 10^9^/L]), all within normal limits. The biochemical panel showed blood urea nitrogen 14 mg/dL (SI: 5.0 mmol/L) (RR: 7-20 mg/dL [SI: 2.5-7.1 mmol/L]), serum creatinine 0.9 mg/dL (SI: 79.6 μmol/L) (RR: 0.6-1.2 mg/dL [SI: 53-106 μmol/L]), alanine aminotransferase (ALT) 28 U/L (RR: 7-56 U/L), aspartate aminotransferase (AST) 24 U/L (RR: 10-40 U/L), alkaline phosphatase 86 U/L (RR: 44-147 U/L), and total bilirubin 0.7 mg/dL (SI: 12.0 μmol/L) (RR: 0.3-1.2 mg/dL [SI: 5.1-20.5 μmol/L]). Serum electrolytes were normal: sodium 139 mmol/L (RR: 135-145 mmol/L), potassium 4.2 mmol/L (RR: 3.5-5.1 mmol/L), chloride 101 mmol/L (RR: 98-107 mmol/L), calcium 9.4 mg/dL (SI: 2.35 mmol/L) (RR: 8.6-10.2 mg/dL [SI: 2.15-2.55 mmol/L]), and magnesium 2.0 mg/dL (SI: 0.82 mmol/L) (RR: 1.7-2.2 mg/dL [SI: 0.70-0.91 mmol/L]).

The endocrine evaluation showed thyroid-stimulating hormone (TSH) 2.1 μIU/mL (RR: 0.4-4.5 μIU/mL), free thyroxine (fT_4_) 1.1 ng/dL (SI: 14.2 pmol/L) (RR: 0.8-1.8 ng/dL [SI: 10.3-23.2 pmol/L]), and HbA1c 5.4% (RR: <5.7%). To evaluate for hypercortisolism, a 24-hour urine free cortisol was obtained; the result was 42 μg/24 hours (SI: 116 nmol/24 hours) (RR: <50 μg/24 hours [SI: <138 nmol/24 hours]), collected in a volume of 1800 mL with a urine creatinine of 1.2 g/24 hours, effectively excluding Cushing syndrome. Inflammatory markers, including C-reactive protein 2.1 mg/L (RR: <5 mg/L) and erythrocyte sedimentation rate 10 mm/hr (RR: <20 mm/hr), were normal.

Spirometry revealed normal lung function: forced expiratory volume in one second (FEV1), 98% predicted; forced vital capacity (FVC), 101% predicted; and FEV1/FVC ratio, 0.82 (RR: ≥0.70). Total lung capacity was normal (80-120% predicted range), and the diffusing capacity of the lungs for carbon monoxide (DLCO) was 96% predicted. These findings excluded obstructive and restrictive lung diseases.

Chest radiography findings were unremarkable. High-resolution computed tomography (CT) of the chest revealed no parenchymal, airway, or interstitial abnormalities.

Electrocardiography demonstrated a normal sinus rhythm. Transthoracic echocardiography showed normal left and right ventricular function, with no evidence of pulmonary hypertension.

Overnight monitoring was performed using transcutaneous carbon dioxide (CO_2_) capnography, which is the preferred noninvasive method for detecting sustained nocturnal hypercapnia. This demonstrated sustained nocturnal hypercapnia throughout the sleep period without obstructive or central apneic events. The calculated AHI was <5 events/hour, effectively excluding obstructive and central sleep apneas. Continuous ventilator-derived end-tidal CO_2_ monitoring was also performed during a period of noninvasive mask-based (bilevel positive airway pressure [BiPAP]) ventilation and demonstrated persistently elevated CO_2_ values correlating with arterial blood gas (ABG)-confirmed arterial hypercapnia, without episodic fluctuations. The convergence of these findings supported sustained alveolar hypoventilation attributable to impaired central ventilatory drive rather than sleep-disordered breathing.

No formal toxicologic urine drug screen for sedatives or opioids was performed; however, the patient repeatedly denied use of such substances, and his medication list was verified as semaglutide only. Although the absence of a formal drug screen is a limitation, the clinical course—with complete resolution after semaglutide discontinuation and no re-exposure to any agent—is consistent with a drug-specific effect.

Application of the Naranjo criteria yielded a score of 6 (probable adverse drug reaction): temporal relationship between drug escalation and symptom onset (+2); improvement after semaglutide discontinuation (+1); no alternative explanation identified (+2); prior literature report absent (0); objective confirmation of hypercapnia with ABG (+1). Rechallenge was not performed because of ethical concerns, which precluded additional points.

The temporal relationship between semaglutide dose escalation and symptom onset, combined with the exclusion of all conventional causes of hypercapnia, strongly suggested medication-induced suppression of central respiratory drive.

## Treatment

Semaglutide was discontinued immediately. The patient was advised to avoid medications or substances known to suppress respiratory drive. Serial ABG monitoring was performed. Due to symptomatic hypercapnia with PaCO_2_ 56 mmHg, a trial of nocturnal noninvasive ventilation (NIV) via BiPAP mask interface was initiated. Multidisciplinary management involved consultation with pulmonology and endocrinology services.

## Outcome and follow-up

Within 10 days of discontinuing semaglutide, the patient reported marked improvement in alertness with complete resolution of morning headaches. Repeat ABG at three weeks demonstrated normalization of PaCO_2_ to 44 mmHg (RR: 35-45 mmHg) and pH to 7.38. NIV was discontinued at this point. At the 3-month follow-up visit, repeat ABG was performed and confirmed sustained normalization (pH 7.39, PaCO_2_ 43 mmHg). The patient remained asymptomatic with no recurrence of hypercapnia and did not require further ventilatory support.

## Discussion

This report describes a previously uncharacterized association between semaglutide use and reversible hypercapnic respiratory failure due to apparent suppression of the central ventilatory drive. The patient presented with excessive daytime sleepiness, morning headaches, and hypercapnia (PaCO_2_ 56 mmHg) with respiratory acidosis, occurring four weeks after dose escalation to 1 mg/week semaglutide [1, 2]. The temporal relationship between medication initiation, dose escalation, symptom onset, and complete resolution within three weeks of discontinuation strongly suggests a causal association. This finding has important implications for the millions of patients currently receiving GLP-1 receptor agonist therapy worldwide [2].

The mechanism underlying semaglutide-induced respiratory depression likely involves GLP-1 receptor-mediated effects on brainstem respiratory control centers. GLP-1 receptors are abundantly expressed in the nucleus tractus solitarius, area postrema, and ventrolateral medulla—regions integral to autonomic regulation and respiratory control—as well as in pulmonary tissue and glandular epithelium [9-11]. Pharmacological doses of semaglutide may attenuate the chemoreceptor response to rising PaCO_2_ levels, thereby reducing the ventilatory drive. Whether this represents a class effect of GLP-1 receptor agonists or is specific to the pharmacokinetic profile of semaglutide (long half-life, high receptor affinity) remains unknown and warrants further investigation. The dose-dependent nature, as evidenced by symptom onset following escalation to 1 mg/week, further supports this hypothesis.

A critical diagnostic challenge was differentiation from obesity hypoventilation syndrome (OHS), defined as daytime hypercapnia (PaCO_2_ > 45 mmHg) in patients with obesity (BMI ≥30 kg/m^2^) with sleep-disordered breathing [12, 13]. Several features argued against primary OHS: transcutaneous CO_2_ overnight monitoring with AHI <5 events/hour excluded sleep-disordered breathing; the acute onset over days rather than insidious progression was atypical; and rapid resolution following drug discontinuation contrasts with OHS, which typically requires ongoing ventilatory support and weight reduction [12]. The precise temporal correlation with medication dose escalation provided a clear precipitating event, which was absent in primary OHS. Longitudinal weight data in this case did not demonstrate significant weight change over the 12-week semaglutide course, making obesity-driven OHS less plausible as the sole explanation.

The systematic evaluation excluded all conventional causes of hypercapnia. Normal spirometry (FEV_1_/FVC 0.82, FEV_1_ 98% predicted) excluded obstructive disease; normal total lung capacity excluded restrictive pathology; normal DLCO (96% predicted) and high-resolution CT excluded parenchymal disease [14]. Neuromuscular causes were excluded by the absence of clinical weakness and the pattern of sustained rather than episodic hypercapnia. Central nervous system structural pathology was unlikely, given the absence of focal neurological findings, acute onset coinciding with medication adjustment, and rapid reversibility. Normal thyroid-stimulating hormone (TSH), free thyroxine (fT_4_), and 24-hour urine free cortisol excluded hypothyroidism and Cushing syndrome [15]. The absence of a formal toxicologic drug screen for opioids or sedatives is acknowledged as a limitation; however, the complete resolution following semaglutide discontinuation—without any other intervention—supports semaglutide as the causative agent.

This case raises important questions regarding patient selection and monitoring during GLP-1 receptor agonist therapy. Patients with preexisting respiratory conditions (chronic obstructive pulmonary disease [COPD], OHS), baseline hypercapnia, or brainstem pathology may warrant enhanced surveillance [14, 15]. The dose-dependent onset indicates that careful titration per recommended schedules and patient education regarding warning symptoms (excessive somnolence, morning headaches) are critical [2]. Clinicians should maintain a low threshold for ABG analysis in individuals who develop unexplained fatigue or excessive somnolence during GLP-1 receptor agonist therapy.

To the best of our knowledge, this is among the earliest reported cases of central respiratory depression associated with a GLP-1 receptor agonist. Future research should include systematic assessment of ventilatory function in ongoing GLP-1 trials, particularly following dose escalation; preclinical studies examining GLP-1 receptor effects on brainstem respiratory centers and chemoreceptor sensitivity [9, 10]; pharmacovigilance database analysis to determine the true incidence; and investigation of genetic polymorphisms predisposing individuals to this adverse effect.

## Learning points

- Semaglutide and other GLP-1 receptor agonists may be associated with rare, reversible hypercapnic respiratory failure likely mediated by central ventilatory drive suppression, possibly through GLP-1 receptor activity in brainstem respiratory centers.
- New-onset somnolence, morning headaches, or fatigue after GLP-1 receptor agonist dose escalation should prompt evaluation for hypercapnia, even in the absence of underlying lung disease.
- Overnight transcutaneous CO_2_ monitoring with AHI calculation is essential to exclude sleep-disordered breathing and confirm the pattern of sustained central hypoventilation.
- Drug discontinuation with supportive NIV can result in rapid and complete resolution of symptoms and ABG abnormalities.

## Contributors

All authors made individual contributions to the authorship. SP, AA: were directly involved in the diagnosis and management of this patient, including clinical assessment and follow-up. LCK: contributed to the physiological and mechanistic interpretation of the findings and provided critical intellectual content. SS, VP: contributed to literature review, data organization, and clinical documentation review. SA: provided overall clinical supervision and critical revision of the manuscript for important intellectual content. All authors reviewed and approved the final draft.

## Data Availability

Original data generated and analyzed for this case report are included in this published article.
